# Optimising Outcomes for Glioblastoma through Subspecialisation in a Regional Cancer Centre

**DOI:** 10.3390/brainsci8100186

**Published:** 2018-10-15

**Authors:** Michael Back, Dasantha Jayamanne, Nicola Cove, Helen Wheeler, Mustafa Khasraw, Linxin Guo, Jemimah Back, Matthew Wong

**Affiliations:** 1 Northern Sydney Cancer Centre, Royal North Shore Hospital, St Leonards, NSW 2065, Australia; 2 Central Coast Cancer Centre, Gosford Hospital, Gosford, NSW 2250, Australia; 3 Genesis Cancer Care, Sydney, NSW 2015, Australia; 4 Sydney Medical School, University of Sydney, Sydney, NSW 2050, Australia; 5 The Brain Cancer Group, Sydney, NSW 2065, Australia; 6 Sydney Brain Tumour Clinic, Sydney, NSW 2065, Australia

**Keywords:** glioblastoma, sub specialization, provider volume

## Abstract

Delivery of highly sophisticated, and subspecialised, management protocols for glioblastoma in low volume rural and regional areas creates potential issues for equivalent quality of care. This study aims to demonstrate the impact on clinical quality indicators through the development of a novel model of care delivering an outsourced subspecialised neuro-oncology service in a regional centre compared with the large volume metropolitan centre. Three hundred and fifty-two patients with glioblastoma were managed under the European Organisation for Research and Treatment of Cancer and National Cancer Institute of Canada Clinical Trials Group (EORTC-NCIC) Protocol, and survival outcome was assessed in relation to potential prognostic factors and the geographical site of treatment, before and after opening of a regional cancer centre. The median overall survival was 17 months (95% CI: 15.5–18.5), with more favourable outcome with age less than 50 years (*p* < 0.001), near-total resection (*p* < 0.001), Eastern Cooperative Oncology Group (ECOG) Performance status 0, 1 (*p* < 0.001), and presence of O-6 methylguanine DNA methyltransferase (MGMT) methylation (*p* = 0.001). There was no difference in survival outcome for patients managed at the regional centre, compared with metropolitan centre (*p* = 0.35). Similarly, no difference was seen with clinical quality process indicators of clinical trial involvement, rates of repeat craniotomy, use of bevacizumab and re-irradiation. This model of neuro-oncology subspecialisation allowed equivalent outcomes to be achieved within a regional cancer centre compared to large volume metropolitan centre.

## 1. Introduction

Since the publication of the European Organisation for Research and Treatment of Cancer and National Cancer Institute of Canada Clinical Trials Group (EORTC-NCIC) Phase III Trial, in 2005 [1], there has been a greater emphasis on optimizing outcome. There has also been more focus on research in patients with high grade glioma, not only regarding systemic chemotherapy, but also with neurosurgical care, radiation therapy (RT) delivery, and diagnostic procedures. This study, updated in 2009, demonstrated that the addition of an oral chemotherapy drug, Temozolomide (TMZ), to standard RT resulted in an increase of median survival from 10.8 months to 14.6 months, and a doubling of 2-year survival [2]. Subsequent improvements in management, with more aggressive guided neurosurgical resection, sophisticated RT techniques, such as intensity modulated radiation therapy (IMRT), exploration of molecular prognostic factors, and targeted therapies, have further improved the median survival of patients [3,4]. Quality care, with specialised neuro-oncology tumour boards, molecular analysis, novel diagnostic MRI-PET imaging, and demand for more tumour specific supportive care services, has increased the infrastructure demands required to manage a comprehensive neuro-oncology practice. This may not be reproducible or efficient resource allocation for low population centres, such as rural or regional areas.

This study explores the outcomes for glioblastoma following the development of a highly-subspecialised model of care for a neuro-oncology service in a regional area of Australia utilising outsourced resources linked to a large metropolitan centre.

## 2. Methods

Newly diagnosed adult patients with a primary brain tumour, referred to the Neuro-oncology Multidisciplinary Tumour board at the Northern Sydney Cancer Centre from March 2008–March 2018, were entered into a prospective database, and approved by Institutional Ethics Review Board. Patients with glioblastoma (World Health Organisation Grade IV glioma) managed with definitive or adjuvant chemoradiation consistent with the EORTC-NCIC protocol [1] were formally included for this study. All patients were managed by one subspecialised radiation oncologist as part of a multidisciplinary neuro-oncology tumour (MDT) team. Patient, tumour, and treatment factors were recorded into a prospective database. Performance status was assessed at the start of RT with the Eastern Cooperative Oncology Group (ECOG) scale.

### 2.1. Neurosurgical Management

Patients were referred from multiple metropolitan neurosurgical units, with an emphasis on optimising extent of resection through utilising techniques including endoscopic surgery and awake craniotomy. Initial diagnostic imaging techniques were centralised at the neurosurgical unit. MRI scans were performed pre-operatively, and generally in the 48-h post-operative period. Metabolic imaging with FET/FDG scans were utilised for patients with MRI demonstrating multifocal areas of enhancement or a suspicious region of T2Flair/non-enhancing T1 hypodensity.

### 2.2. Neuropathological Features

All patients had the diagnosis of WHO 2007 Grade IV glioma confirmed by standard immunohistochemical techniques [5]. The proliferation index, Ki67 percentage positive (Ki67%), was recorded. Potential prognostic molecular profiles using protein sequencing for isocitrate dehydrogenase (IDH) mutation, O-6-methylguanine-DNA methyltransferase (MGMT) promoter status and EGFR amplification were gradually introduced into practice from 2012 as evidence improved for their utility [6].

### 2.3. Radiation Therapy

Patients were managed under a centralised protocol from the Neuro-Oncology MDT with dose fractionation schedules as per the EORTC-NCIC protocol with 60 Gy being delivered in 30 fractions over a 6-week period [1]. Treatment technique was with IMRT and image guided radiation therapy in all patients, commencing between day 21 and day 28 post-craniotomy.

### 2.4. Systemic Therapy

Systemic management followed the EORTC-NCIC protocol regimen with TMZ used in two phases: Initially at 75 mg/m^2^ daily during the RT, followed by a 4-week break; then 150–200 mg/m^2^ for days 1–5 every 28 days for 6 months [1]. During the study time period, patients may have been enrolled in one of eight multicentre adjuvant therapy clinical trials (Phase III Merck Serono CENTRIC with Celingitide [7]; Phase III Roche AVAglio with Bevacizumab [8]; Phase II Merck Serono ExCENTRIC with Celingitide and procarbazine [9]; Phase III Cell- Dex ACT IV with Rindopepimut a EGFRvIII monoclonal antibody [10]; Randomised Phase II VERTU Study with veliparib [11]; Phase III ABT414 for EGFR amplified glioblastoma [12]; Randomised Checkmate Nivolumab studies for both MGMT methylated and unmethylated GBM [13]).

### 2.5. Model of Care for Adjuvant Therapy

All patients had management decision-making centralised at the Neuro-Oncology MDT at the Northern Sydney Cancer Centre (NSCC), a metropolitan based tertiary hospital in Sydney. All adjuvant therapy between March 2008 and March 2013 was delivered at this institution.

In March 2013, a regional cancer centre (Central Coast Cancer Centre), situated 70 km north of the NSCC, was opened providing generalist independent radiation oncology services for a population of 330,000 [14]. This region has no intracranial neurosurgical facilities in either the public or private sector, with newly diagnosed patients with brain tumours being transferred to the metropolitan centre.

Recognising the subspecialised nature of neuro-oncology services and the absence of local neurosurgical services, a decision was made to appoint a lead clinician (radiation oncologist) from the metropolitan Neuro-Oncology MDT to manage and organise patient care on-site at the regional centre. This role was as a visiting medical officer with attendance at a 0.2 full-time equivalent. Supportive services were then mobilised from existing medical oncology and cancer nurse co-ordinator resources on site at the regional centre. All patients had case review and discussion through the centralised metropolitan Neuro-Oncology MDT, but then with therapy directed and delivered at the regional centre by the lead clinician in combination with the local teams. At any relapse or clinical event the imaging review and discussion was noted at the metropolitan Neuro-Oncology MDT.

### 2.6. Follow-Up

All patients were followed closely with initial MRI at 1-month post-RT (M + 1) then second monthly MRI until completion of adjuvant TMZ, then three monthly until end of year 3 post-RT, then four to six monthly until progression. Features, suggestive of pseudoprogression, were actively investigated to exclude the risk of true relapse. Salvage treatments were individualised based on the extent of disease and patient factors, but may include repeat craniotomy, second to third line cytotoxic chemotherapy, targeted therapy with bevacizumab (BEV), re-irradiation or supportive care only.

### 2.7. Clinical Quality Indicators

Four geographical treatment groups were created based on the criteria of treatment based at metropolitan centre or regional centre; and being managed before and after the March 2013 opening of the regional cancer centre. Groups 1 and 3 were from the metropolitan centre, and were pre-2013 (Group 1) and post-2013 (Group 3). Groups 2 and 4 were residents of the regional area and were managed pre-2013 at the metropolitan centre (Group 2) and post-2013 at the regional cancer centre (Group 4).

Clinical quality indicators were developed to demonstrate any difference between the Groups. These were chosen as reflecting high quality or subspecialised neuro-oncology care and involved clinical outcome indicators (overall survival and relapse free survival), and process indicators (Clinical trial involvement, rate of repeat craniotomy, use of BEV and use of re-irradiation).

### 2.8. Statistical Considerations

The primary endpoint was the overall survival time in months calculated from the time of initial surgical diagnosis of WHO Grade IV glioma to death or date of censure on 31 August 2018. Survival curves were generated using Kaplan Meier method. Univariate predictors of survival duration were evaluated using log-rank comparisons. All reported *p*-values are two-tailed. Statistical significance was defined as *p* < 0.05 in all cases. IBM SPSS Statistics version 23 (IBM Corporation, Armonk, NY, USA) was used for statistical analysis.

## 3. Results

Three hundred and fifty-two consecutive patients with WHO Grade IV glioma, managed with intensity modulated RT and TMZ following the EORTC-NCIC protocol between 1 March 2008 and 1 March 2018, were identified from the database and included for analysis. A minimum of six months follow-up was present for all surviving patients. Patient and tumour characteristics are summarised in Table 1.

The median age of patients at diagnosis was 57 years with 24% patients aged 50 years or younger. ECOG Performance status at commencement of RT was 0–1, and 2–3, in 71%, and 29%, of patients, respectively. Temporal lobe (31%) and frontal lobe (29%) were the most frequent neuroanatomical sites. A near total surgical resection was performed in 44%, subtotal in 40%, and 16% had biopsy only. Unfortunately MGMT promoter methylation was only performed in 50% of patients and methylation status was positive in 45% of those patients tested. All patients were managed with IMRT and received TMZ concurrently during the RT. Although accrual for clinical trials varies as to eligibility criteria (such as MGMT methylation) and the availability of trials, 22% patients were managed under one of the eight multicentre clinical trials.

For the geographical location, 238 patients were resident in metropolitan area (Groups 1 and 3) and 114 in regional area (Groups 2 and 4). Prior to March 2013, the 44 regional patients (Group 2) were managed at the metropolitan centre and then subsequently the 70 regional patients (Group 4) were managed at the regional centre.

### 3.1. Relapse Free Survival

Two hundred and ninety-four patients have relapsed, with a median relapse—free survival of 11 months (95% CI: 10.0–12.0). The pattern of relapse included a component of local failure in 68% of relapses, marginal failure in 10% and distant failure in 29%.

### 3.2. Overall Survival

Two hundred and eighty-four patients are deceased, with a median overall survival of 17 months (95% CI: 15.5–18.5). Twelve patients were deceased without confirmation of relapse, though eight were being managed for pseudoprogression. The actuarial rate of overall survival at 12, 24 and 36 months of 78%, 32% and 22% respectively.

Evaluation of potential prognostic factors demonstrated that survival was associated with age less than 50 years (*p* < 0.001), near total resection (*p* < 0.001), ECOG PS 0, 1 (*p* < 0.001), and MGMT Methylation status (*p* = 0.001). Year of diagnosis cohorts, neuroanatomical site of tumour, and Ki67% level were not associated with survival.

Specifically, for patients with a near-total resection the median survival was 21.0 months (95% CI: 18.6–23.4), compared to subtotal resection of 16.0 months (95% CI: 14.7–17.3), and biopsy only of 13.0 months (95% CI: 11.0–15.0). In the population of patients with known molecular factors patients the median survival in those with an IDH mutated GBM was 56.0 months (95% CI: 32.5–79.5); whilst those with MGMT methylation was 28.0 months (95% CI: 17.6–38.4).

### 3.3. Salvage Therapy

Of the 294 patients with evidence of relapse, 97% patients were managed with at least one course of salvage chemotherapy. Additionally, 35% had a further craniotomy at time of relapse. Patients relapsing after March 2011 had the potential for salvage therapy utilising BEV, as well as use of re-irradiation. Of the 227 relapses since that date, 73% had BEV and 21% received re-irradiation.

### 3.4. Analysis of Geographical Location Groups

**Figure 1:**
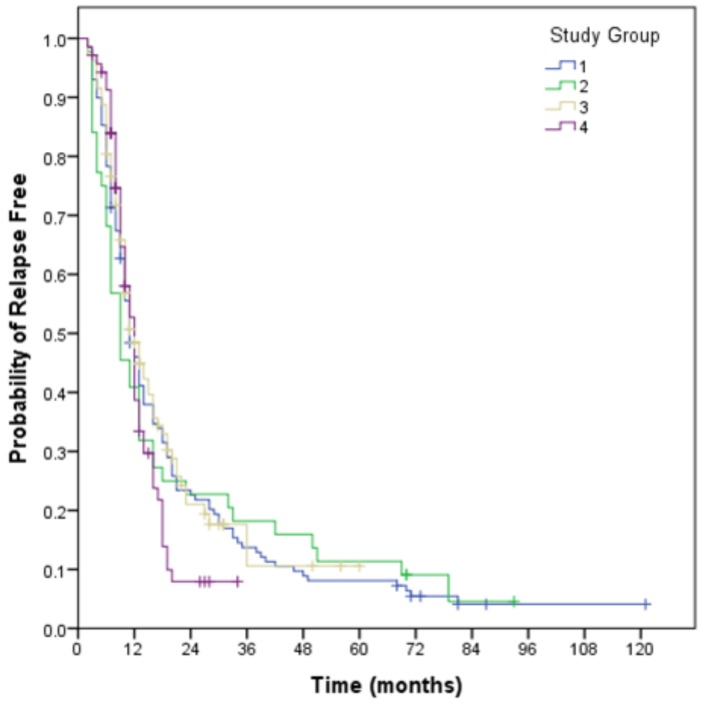
Relapse free survival for geographical Groups 1–4. (Group 1: Metropolitan centre pre-2013; Group 2: Regional residents managed pre-2013 at the metropolitan centre; Group 3: Metropolitan centre post-2013; Group 4: Regional residents managed post-2013 at the regional centre).

**Figure 2:**
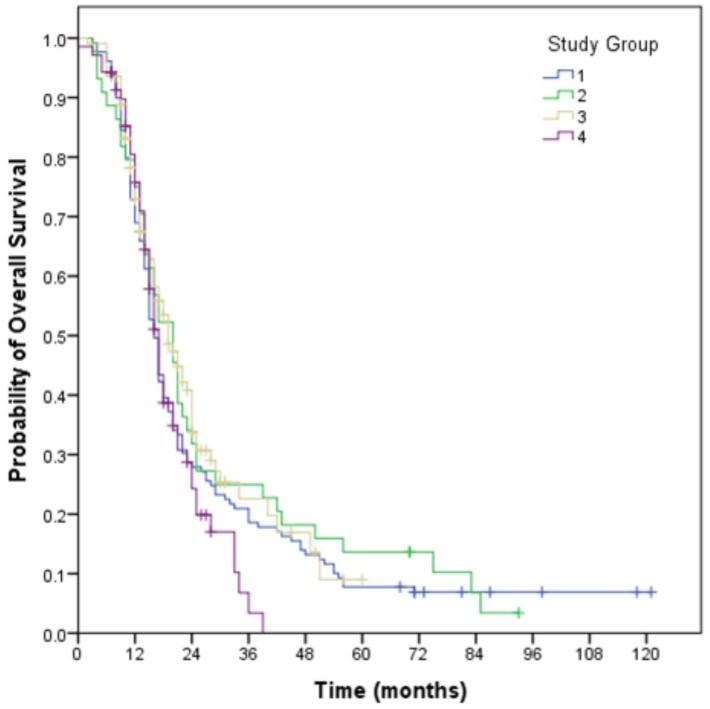
Overall survival for geographical Groups 1–4. (Group 1: Metropolitan centre pre-2013; Group 2: Regional residents managed pre-2013 at the metropolitan centre; Group 3: Metropolitan centre post-2013; Group 4: Regional residents managed post-2013 at the regional centre).

No significance difference overall survival for Groups 1 to 4 with median survivals of 16, 20, 19, and 17 months respectively (*p* = 0.35). Survival curves for both relapse free survival and overall survival for the Groups are demonstrated in Figure 1 and Figure 2. 

The distribution of survival outcomes in Groups for each of the favourable prognostic factors of young age, near-total extent of resection, ECOG Performance status 0, 1 and MGMT methylation are listed in Table 2, confirming equivalent outcome within each of the four patient Groups. In regard to clinical trial involvement there was no difference between patients managed at the regional centre on clinical trials (17% and 18% for Groups 3 and 4); and in the period from 2016 the proportion of patients on trial increased to 32% and 38% respectively once the further protocols were established.

At time of relapse the clinical quality process indicator outcomes were equivalent in regard to utilisation of repeat craniotomy, BEV use and re-irradiation (Table 3).

## 4. Discussion

This study confirms the improved survival of patients managed for glioblastoma in the era post-2006, since the introduction of temozolomide to the adjuvant therapy protocol. Median survival for newly diagnosed patients now approaches 20 months, which is improved from the results obtained from the EORTC-NCI Study of 14.6 months [1]. Increased subspecialisation with sophistication of therapy and supportive care may contribute to these additional benefits in median survival. Additionally, this study demonstrates that a subspecialised neuro-oncology service based at a regional setting managing patients for glioblastoma can be provide high quality care with outcomes for patients with glioblastoma equivalent to a comprehensive metropolitan cancer centre. Seamless pathways were produced, and patients had access to innovative highly sophisticated therapies and clinical trials. 

The opening of cancer centres in regional areas aims not only provide geographical access to specialised cancer therapy (improving access gaps), but also counter the discrepancies in cancer outcomes demonstrated by higher mortality related to distance from metropolitan centres [15]. However, the opening of such centres does not necessarily resolve these issues. With increase dispersion of care there may be reduced volume of cases per clinician; and with clinicians depending on a generalist model the low provider volume and lack of subspecialisation may also negatively influence outcomes. 

Neuro-oncology is a low volume subspecialty compared to other cancer subsites. The incidence of high grade glioma is 7.3 cases per 100,000 population per year and accounts for only 1.3% of all cancer diagnoses [16]. With such low volume presenting annually, the ability to establish a local service of expertise is compromised. The impact of case volume per provider has been demonstrated to influence cancer outcomes, most prominent in surgical oncology [17,18,19]. Individual surgeon volume has been demonstrated to impact both on cancer survival, but also treatment related morbidity, especially in colorectal cancer and breast cancer [20]. Additionally, adverse outcomes have been noted in other cancer subsites with actual institution volume suggestive that it may be related to the pathway of care along the whole patient journey in low volume services [20].

In radiation oncology, there is less data available, however adverse outcome has been demonstrated for lower volume clinician or institutional providers in the management of head and neck cancer and lung cancer [21,22,23]. Radiation oncologist experience was noted in a US study to adversely impact on outcome in patients managed with IMRT, a sophisticated treatment approach in head and neck cancer [21]. This study compared clinician outcome at the median number of procedures performed. With 15 more patients per clinician there was a 50% relative improvement in survival; whilst 15 fewer patients produced a 76% reduction in survival. This was accounted by the authors through potential errors in target volume delineation. Establishment of centralised protocols has been viewed as a method to overcome this low provider effect, and is a favoured method to improve treatment quality. 

However, the provider effect may extend beyond this aspect, with clinical trial data from the RTOG lung cancer [22] and head and neck cancer [23] trials suggesting that the impact may be related to experience than protocol adherence. RTOG 0617 study on concurrent chemoradiation therapy for locally advanced non-small cell lung cancer demonstrating a 10% absolute worse survival for patients managed at low volume institutions, despite there being no difference in clinical trial protocol violations [22]. This difference was accounted for by improved treatment design and better management of adverse effects in the larger volume institutions. one approach described by the authors may be to concentrate patient care and clinical trial participation to high-volume centres, however this would potentially discriminate against patient participation from rural care centres and limit access to novel or improved therapies. In the current study this would work against the access block that the development of regional cancer centres aimed to overcome. 

RTOG 0129 examining altered fractionation regimen in chemoradiation therapy for head and neck cancer similarly demonstrated an adverse effect on survival of accrual volume with 51.0% vs. 69.1% 5-year survival with historically low versus high volume RTOG Trial accrual institutions [23]. Whilst unacceptable deviations from protocol independently increased the risk of death and were seen more frequently at low accrual centres, the authors only accounted 21% of the adverse survival difference to the protocol deviation. Conclusions from the authors included recommendations that subspecialisation should be promoted by cancer centres and training programmes; as well as improved access for patients to oncologists who specialise in head and neck cancer and manage patients at high volume centres.

The inference from these studies is that the benefit of a high-volume clinician provider is not related just to radiation therapy protocol adherence, but the whole patient journey. In glioblastoma this decision-making needs to be individualised at times of follow-up based on issues of MRI interpretation, management of pseudoprogression, adjustment of corticosteroids and implementation of salvage therapies. With the complexity of management and patient care, there is a need for neurological subspecialised skills, either as a neuro-oncologist or co-ordination of neurology expertise with interest in brain tumours. 

For the regional cancer centre in this current study, the management of a low volume neuro-oncology service could have been undertaken in three models of care. Firstly, there could be an independent centre model where an interested, but not subspecialised, clinician would manage the patients. Ideally this would involve educational links with a major centre. Secondly a mentorship model where the metropolitan centre conducts a regional outreach clinic, but then manages the patient centrally back at the metropolitan centre. The third option, which was implemented, involved the development of the subspecialty model where a high-volume specialist from the metropolitan centre manages the patient care directly at the regional centre, establishing a local team that is linked to the centralised service. This involved the role of a lead clinician, development of collaborative clinicians on site, seamless digital imaging links between central and regional centres, ability to admit patients at both sites to facilitate prompt neurosurgical review or subspecialised investigations, and education sessions at the regional centres for others involved in the patient pathways. This in then allowed not only protocol adherence, but also adoption of the process indicator endpoints facilitating good clinical quality and subsequent outcome.

For future patients, the demonstration of equivalent clinical quality outcomes can provide both confidence in care at the regional centre, but also provide the framework for expansion of the subspecialised service. This will allow the implementation of innovative therapies that can hopefully reduce the disease burden caused by high grade glioma.

## 5. Conclusions

This study confirms that the significant improvement in median survival for glioblastoma that has occurred in recent years following the introduction of a multidisciplinary approach with chemoradiation therapy can be translated to a regional cancer centre. A model incorporating a subspecialised neuro-oncology service with an outsourced high-volume clinician can deliver a high quality service with equivalent outcomes to a large metropolitan centre.

## Figures and Tables

**Table 1:** Patient and treatment characteristics.

	Subgroup	Number (352)
Age at Diagnosis	<50 years >50 years Median	86 (24%) 266 (76%) 43 years
Year of Diagnosis	2008–2010 2011–2014 2015–2018	109 (31%) 125 (36%) 118 (33%)
Tumour Site	Temporal Frontal Parietal Occipital Thalamic Other	110 (31%) 101 (29%) 89 (25%) 26 (7%) 20 (6%) 6 (2%)
Extent of Resection	Near-Total Subtotal Biopsy	156 (44%) 142 (40%) 54 (16%)
Ki67%	<20 20–30 31–50 >50 Unknown	48 (14%) 122 (35%) 93 (26%) 49 (14%) 40 (11%)
Isocitrate Dehydrogenase (IDH) Mutation	Wildtype Mutation Unknown	244 (69%) 17 (5%) 91 (26%)
O-6 methylguanine DNA methyltransferase (MGMT) Methylation	No Yes Unknown	97 (28%) 79 (22%) 176 (50%)
Eastern Cooperative Oncology Group (ECOG) PreRT	0 1 2 3, 4	104 (29%) 146 (41%) 75 (21%) 27 (8%)

**Table 2:** Survival for favourable prognostic factors within geographical Groups 1–4.

	Subgroup	All Patients 2008–2018 (352)	Group 1 Metro 2008–2013 (129)	Group 2 Regional 2008–2013 (44)	Group 3 Metro 2013–2018 (109)	Group 4 Regional 2013–2018 (70)
Age <50 years	Median (months) 95% CI	24 months (19.3–28.7)	23 (13.9–32.1)	NA	24 (20.5–27.5)	16 (12.8–19.2)
Extent of Resection (Near Total)	Median (months) 95% CI	21 months (18.6–23.4)	19 (15.3–22.7)	22 (18.5–25.5)	21 (15.6–26.4)	20 (15.4–26.4)
Performance Status (ECOG 0, 1)	Median (months) 95% CI	20 months (18.0–22.0)	17 (14.7–19.3)	21 (17.1–24.9)	23 (20.2–25.8)	18 (15.1–20.9)
MGMT (Methylated)	Median (months) 95% CI	28 months (17.6–38.4)	36 (10.2–61.8)	NA	28 (19.8–36.2)	20 (13.3–20.0)

**Table 3:** Clinical Quality Indicators for geographical Groups 1–4.

	Outcome	All Patients 2008–2018 (352)	Group 1 Metro 2008–2013 (129)	Group 2 Regional 2008–2013 (44)	Group 3 Metro 2013–2018 (109)	Group 4 Regional 2013–2018 (70)
Overall Survival	Median (months) 95% CI	17 (15.5–18.5)	16 (14.5–17.5)	20 (16.0–24.0)	19 (15.3–22.7)	17 (15.4–18.6)
Relapse Free Survival	Median (months) 95% CI	11 (10.0–12.0)	11 (9.2–12.8)	9 (5.3–12.7)	12 (9.6–14.4)	12 (10.7–13.3)
Clinical Trial Accrual	% involved	22%	29%	18%	17%	18%
Repeat Craniotomy at Salvage	% use	35%	30%	32%	43%	31%
Bevacizumab Use at Salvage	% use post 2011	73%	65%	48%	58%	73%
Re-irradiation at Salvage	% use post 2011	21%	16%	14%	20%	24%
